# Functional Interactions between Retinoblastoma and c-MYC in a Mouse Model of Hepatocellular Carcinoma

**DOI:** 10.1371/journal.pone.0019758

**Published:** 2011-05-06

**Authors:** Louis A. Saddic, Stacey Wirt, Hannes Vogel, Dean W. Felsher, Julien Sage

**Affiliations:** 1 Department of Pediatrics, Stanford University, Stanford, California, United States of America; 2 Department of Genetics, Stanford University, Stanford, California, United States of America; 3 Department of Pathology, Stanford University, Stanford, California, United States of America; 4 Department of Medicine, Stanford University, Stanford, California, United States of America; Ulm University, Germany

## Abstract

Inactivation of the RB tumor suppressor and activation of the MYC family of oncogenes are frequent events in a large spectrum of human cancers. Loss of RB function and MYC activation are thought to control both overlapping and distinct cellular processes during cell cycle progression. However, how these two major cancer genes functionally interact during tumorigenesis is still unclear. Here, we sought to test whether loss of RB function would affect cancer development in a mouse model of c-MYC-induced hepatocellular carcinoma (HCC), a deadly cancer type in which RB is frequently inactivated and c-MYC often activated. We found that RB inactivation has minimal effects on the cell cycle, cell death, and differentiation features of liver tumors driven by increased levels of c-MYC. However, combined loss of RB and activation of c-MYC led to an increase in polyploidy in mature hepatocytes before the development of tumors. There was a trend for decreased survival in double mutant animals compared to mice developing c-MYC-induced tumors. Thus, loss of RB function does not provide a proliferative advantage to c-MYC-expressing HCC cells but the RB and c-MYC pathways may cooperate to control the polyploidy of mature hepatocytes.

## Introduction

Cancer is a complex disease that often progresses slowly due to the gradual accumulation of genetic and epigenetic alterations over time [1], [2]. Typically, tumor cells harbor mutations that activate oncogenes and inactivate tumor suppressors. The combination of these alterations promotes deregulated cell division, one of the hallmarks of the cancer phenotype [1]. Despite this universal property of tumors, many outstanding questions remain, including whether the order of the successive alterations is critical to cellular transformation and how mutations in cancer pathways cooperate in the course of the disease.

The Retinoblastoma protein (RB) is a potent tumor suppressor that restricts S phase entry by inhibiting the activity of the E2F family of transcription factors [3]. Early in G1, activation of Cyclin/CDK complexes by mitogenic signals results in RB phosphorylation and functional inactivation, thus allowing E2F family members to transcribe genes necessary for cell cycle progression [4]. In addition to this well-described function of RB, emerging evidence indicates that RB also normally promotes differentiation in multiple lineages [5], [6], [7], [8], [9], [10], [11] and protects cells from the accumulation of genomic alterations [12], [13], [14], [15], [16], [17]. Due to the critical influence of RB in the control of cell cycle progression, it is not surprising that RB or members of the RB pathway are mutated in nearly all human cancers [18], [19].

c-MYC (hereafter referred to as MYC) is a transcription factor that heterodimerizes with its partner MAX in order to control the expression of a large program of genes that promote proliferation, cell death, cell growth, and cellular differentiation [20], [21], [22], [23]. In resting cells, MYC activity is often minimal because of low mRNA and protein levels; in contrast, MYC activity is strongly induced in tumor cells by multiple mechanisms, including increased transcription, stabilization of the protein, gene amplification, and chromosomal translocation [24], [25]. MYC activation is a common feature of many human cancers, including cancers with mutations in the RB pathway [20], [21], [22], [26].

Hepatocellular carcinoma (HCC) is the third most common cause of cancer death in the world with more than 500,000 deaths a year [27]; the number of HCC cases increases every year [28]. While several causal agents for HCC have been identified, including infection with hepatitis B and C viruses (HBV and HCV), there is no effective treatment for this cancer type, in part because the molecular and cellular mechanisms of HCC development are still poorly understood [29], [30], [31]. MYC is amplified in up to 50% of HCC cases, suggesting a key role for MYC activation in the development of these tumors [32], [33], [34]. Similarly, inactivation of the RB pathway is found in more than two-thirds of human HCCs by several mechanisms, including inhibition of p16^INK4a^ and its family member p15^INK4b^, increased expression of Cyclin D1, and loss of RB function by phosphorylation, protein degradation, or gene mutation [35], [36].

Mouse models carrying mutations commonly found in human tumors provide an opportunity to investigate the mechanisms of tumorigenesis *in vivo*. A mouse model of human HCC with inducible expression of MYC in adult liver cells has shown that overexpression of MYC is sufficient to initiate HCC development; however, these tumors develop with a prolonged latency, suggesting that other genetic alterations are necessary to generate HCC, including mutations in the p53 pathway [32], [37], [38], [39], [40]. The frequency of MYC activation and loss of RB pathway function in human HCC suggests that these two pathways may interact during tumorigenesis in the liver. However, whether or not inactivation of RB can cooperate with MYC in the formation of murine HCC has yet to be demonstrated. In this report, we use a mouse model that allows us to conditionally delete the *Rb* gene and overexpress *MYC* specifically in the liver. We show that loss of RB has minimal effects on the development of HCC initiated by the overexpression of MYC, suggesting that these two cancer genes share many functions in liver cells undergoing tumorigenic transformation.

## Results

### Combined activation of MYC and inactivation of RB in the liver of adult mice results in the development of hepatocellular carcinoma

**Figure 1:**
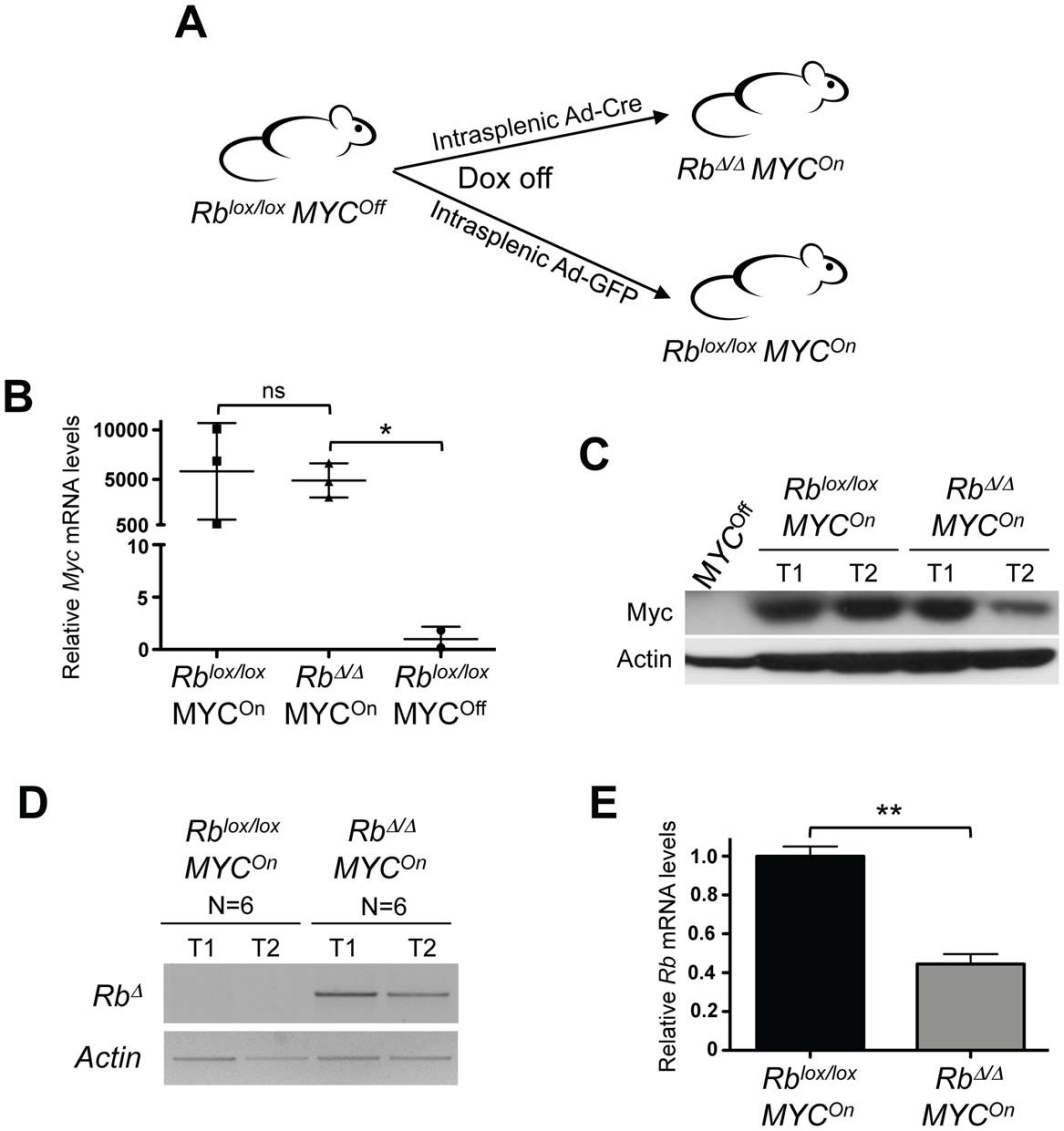
MYC activation and Rb deletion drive HCC development in the liver of adult mice. **A.** Strategy to produce *MYC* and *MYC/Rb* mutant tumors using *Rb^lox/lox^* and TRE-*MYC* LAP-*tTA* mice. MYC is activated in the liver by the removal of doxycycline from the drinking water while *Rb* is specifically deleted in the liver by splenic injection of Ad-Cre. Ad-GFP is used as a control. **B.** RT-qPCR analysis of *MYC* RNA levels in control livers (*Rb^lox/lox^ MYC^Off^*, n = 2) and in *MYC* and *MYC/Rb* mutant tumors (n = 3). **C.** Immunoblot analysis of MYC protein levels in control livers (*MYC^Off^*) and in *MYC* and *MYC/Rb* mutant tumors (two independent tumors each, T1 and T2). Actin serves as a loading control. **D.** Genomic PCR analysis for the deleted allele of *Rb* (*Rb^Δ^*) using DNA from *MYC* and *MYC/Rb* mutant tumors. *Actin* serves as a positive PCR control. **E.** RT-qPCR analysis of *Rb* RNA levels in *MYC* (black, n = 3) and *MYC/Rb* (grey, n = 3) mutant tumors.

To investigate the potential interactions between MYC overexpression and RB loss of function in HCC, we bred conditional mutant *Rb^lox/lox^* mice [41] with *LAP-tTA TRE-MYC* mice, in which expression of a human *MYC* cDNA can be induced specifically in the liver [32], [42] (Figure 1A). We found that, as previously shown [32], [42], expression of MYC in the liver of adult mice (“*MYC* mutant mice”) resulted in the development of liver tumors. We used intrasplenic injection of an adenoviral vector expressing the Cre recombinase (Ad-Cre) to specifically delete *Rb* in the adult liver and found that loss of *Rb* was not sufficient to initiate liver cancer development, as previously shown [43], [44] (data not shown, see below). *MYC/Rb* double mutant mice developed liver tumors composed of multiple fleshy vascular nodules resembling *MYC* mutant tumors (data not shown). Total RNA from tumors macro-dissected at the surface of the liver showed high levels of expression of *MYC* compared to control liver samples, independent of the presence or the absence of *Rb*, as expected (Figure 1B). Immunoblot analysis of liver extracts revealed high levels of the MYC protein in all tumor samples compared to controls (Figure 1C). PCR analysis of genomic DNA isolated from tumors showed deletion of *Rb* in all the double mutant tumors examined (n = 6); no *Rb* deletion was observed in mice expressing the *MYC* transgene and infected with an Ad-GFP control adenovirus (n = 6) (Figure 1D). Quantitative RT-PCR (RT-qPCR) analysis of total RNA extracted from *MYC/Rb* double mutant tumors showed a decrease in *Rb* levels compared to *MYC* mutant tumors (Figure 1E). These data showed that deletion of *Rb* in the liver of the infected mice was efficient and not counter-selected during the development of HCC initiated by activation of MYC.

**Figure 2:**
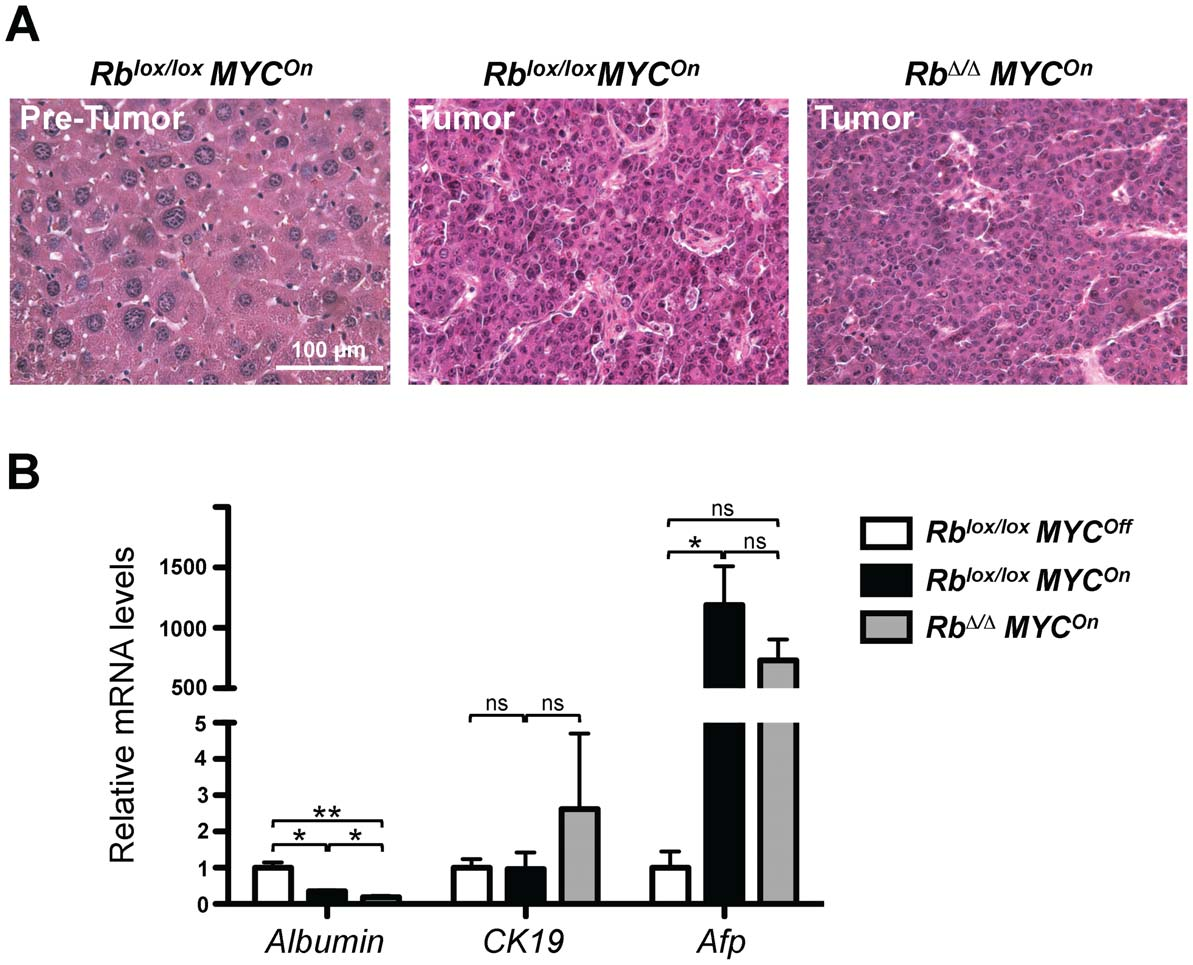
Rb inactivation does not change the histology and differentiation status of MYC-induced HCC. **A.** Histology of non-tumor liver tissue from *MYC* mutant mice (left) and tumor tissue from *MYC* (center) and *MYC/Rb* (right) mutant mice. **B.** RT-qPCR analysis of *Albumin* (a marker of hepatocytes), *CK19* (a marker of bile ducts), and *Afp* (a marker of HCC) in control livers (white, n = 2), *MYC* mutant tumors (black, n = 3), and *MYC/Rb* mutant tumors (grey, n = 3). Note that the p-value for *Afp* between control livers and *MYC/Rb* mutant tumors is 0.06, just below the significance threshold.

When we analyzed livers from *MYC* and *MYC/Rb* mutant mice, we found that tumors from both genotypes were composed of hepatocellular neoplasms characterized by sheets of cells with occasional mitotic figures, slightly pleomorphic nuclei, and prominent nucleoli (Figure 2A). Examples from each genotype showed a range of differentiation between well- and moderately-differentiated HCC without an obvious tendency in the presence of either genotype. In addition, tumors from both genotypes expressed levels of *Albumin*, a marker of hepatocytes, that were lower than those found in control livers. Levels of *Cytokeratin 19* (*CK19*), a marker of cholangiocytes and some liver progenitors [45], were not significantly different between control livers and the tumors. Levels of *Afp* were increased in tumors, similar to what is commonly observed in human HCC [46] (Figure 2B). These observations indicate that loss of *Rb* in this mouse model of HCC induced by expression of MYC does not grossly affect the histopathological features of these tumors, allowing us to further investigate how loss of RB and MYC overexpression may function during HCC development.

### Activation of MYC and loss of RB do not cooperate to control cell cycle progression in the liver of adult mice

**Figure 3:**
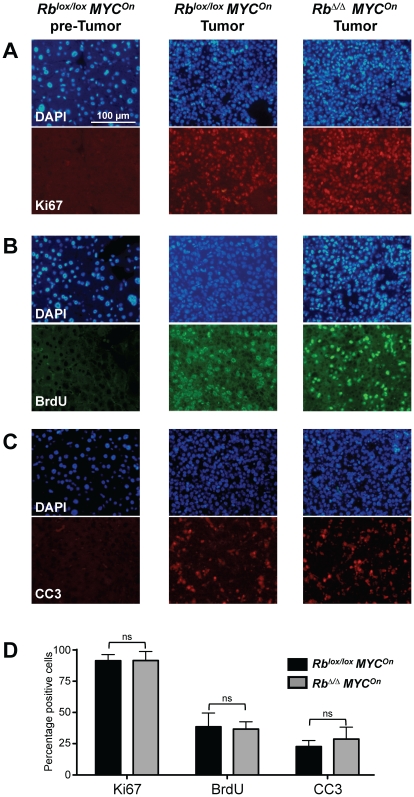
Rb inactivation does not affect the degree of proliferation or cell death of MYC-induced HCC. **A–C.** Immunofluorescence staining for Ki67 (**A**), BrdU (**B**), and cleaved caspase 3 (CC3) (**C**) on *MYC* mutant non-tumor liver tissue and tumor tissue from *MYC* and *MYC/Rb* mutant mice. DAPI nuclear staining is used to indicate the density of cells on the sections. The pictures shown are representative of each group. **D.** Quantification of staining shown in A. The number of positively stained cells for each antibody over the number of DAPI stained cells expressed as a percent was determined using the BioQuant software. For each antibody and genotype combination the average of n = 2 mice was calculated where each n is the average percent of positive cells from three fields containing at least 250 cells each.

**Figure 4:**
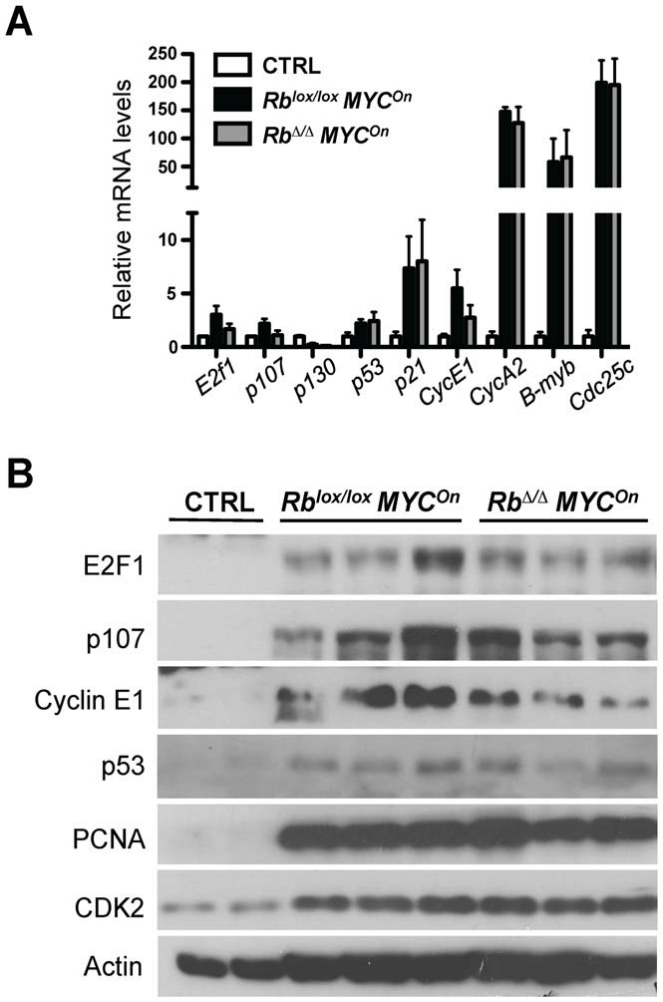
Rb inactivation does not change the mRNA expression or protein levels of cell cycle markers in MYC-induced HCC. **A.** RT-qPCR for several cell cycle genes on RNA from *MYC* (black, n = 3) and *MYC/Rb* (grey, n = 3) mutant HCC. *Rb^lox/lox^ MYC^Off^* non-tumor liver is used as a control (CTRL) (white, n = 2). There is not statistical difference for any of these gene expression levels between *MYC* and *MYC/Rb* tumors. **B.** Representative immunoblot analysis for several cell cycle markers on protein extracts from *MYC* and *MYC/Rb* mutant HCC compared to wild-type liver. The concentration of the protein extracts was quantified using a Bradford assay and similar amounts of proteins were loaded in each lane. Actin serves as a loading control; note that CTRL liver cells express less Actin per µg of protein extract than tumor cells.

Immunofluorescence analysis for Ki67 expression, a marker of cycling cells, and for BrdU incorporation, a marker of DNA replication, showed that tumors from both genotypes displayed high indices of proliferation that were not visibly different (Figure 3A–B and D). *MYC* and *MYC/Rb* mutant tumors also showed grossly similar levels of apoptotic cell death as detected by immunofluorescence for cleaved caspase 3 (CC3) (Figure 3C–D). To corroborate these observations made on tumor sections, we next measured mRNA and protein expression levels of cell cycle genes from dissected tumors. We did not detect any significant differences between *MYC* and *MYC/Rb* mutant tumors, which both expressed high levels of these cell cycle regulators compared to wild-type livers (Figure 4A–B).

**Figure 5:**
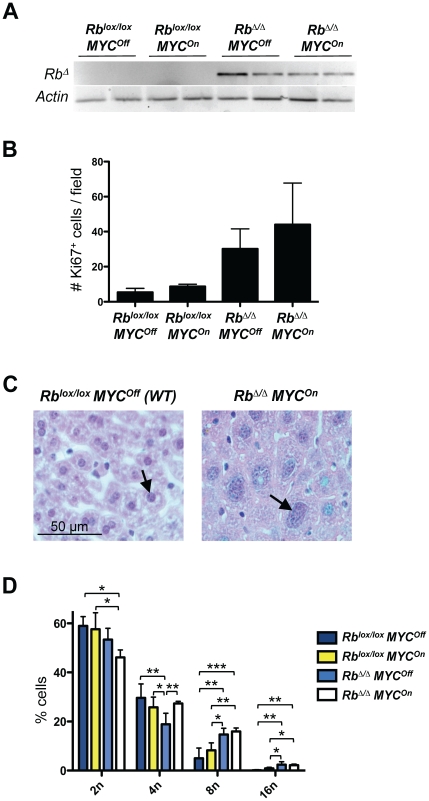
Rb inactivation and MYC activation cooperate to increase ploidy in hepatocytes. **A.** Genomic PCR analysis for the deleted allele of *Rb* (*Rb* ^Δ^) using DNA from control, *MYC*, *Rb*, and *MYC/Rb* mutant livers before the development of tumors in *Rosa26^+/CreER^* mice. *Actin* serves as a positive PCR control. **B.** Quantification of immunostaining experiments for the number of Ki67^+^ liver cells per field on control, *MYC*, *Rb*, and *MYC/Rb* mutant liver sections 5 weeks after the deletion of *Rb* and the induction of *MYC*. The number of positive cells in eight 20× fields was counted in 2 mice for each treatment group. The differences observed are not significant in a t-test. **C.** Representative microphotographs of hepatocytes from a mouse injected with corn oil alone in the presence of doxycycline (wild-type for RB and MYC) and a mouse injected with tamoxifen in the absence of doxycycline are shown (*MYC/Rb* mutant liver). Arrows show a small nucleus in the control mouse and a large nucleus in the mutant mouse. **D.** Quantification of ploidy by FACS of hepatocyte populations from control (n = 5), *MYC* (n = 8), *Rb* (n = 5), and *MYC/Rb* (n = 8) mutant livers 5 months after *Rb* deletion (*Rosa26^+/CreER^* mice) and MYC activation. Only statistically significant differences are shown.

These observations indicated that *MYC* and *MYC/Rb* mutant HCCs were very similar but did not exclude that the early stages of cancer development may have different characteristics. To begin to investigate the features of liver cancer initiation in mice from both genotypes, *LAP-tTA TRE-MYC* mice were bred to *Rosa26^+/CreER^ Rb^lox/lox^* mice, in which recombination of alleles flanked by *lox* sites can be triggered by administration of intraperitoneal injections of tamoxifen. We found that this approach gave less variable results than the adenoviral infection at early time points (data not shown) and resulted in the efficient deletion of *Rb* (Figure 5A).

Previous studies have shown that activation of the *MYC* transgene in the liver of adult mice does not change the proliferation of hepatocytes compared to wild-type livers [32], [42]. In contrast, deletion of *Rb* is not sufficient to initiate liver cancer development but has been shown to cause widespread cell cycle-entry in hepatocytes [44]. We observed that, before any tumor lesions could be observed histopathologically, *Rb* mutant mice displayed elevated Ki67 staining on liver sections compared to *Rb* wild-type mice; activation of the *MYC* transgene did not induce proliferation in populations of adult hepatocytes, as expected, and slightly increased the number of Ki67^+^ cells in *Rb* deficient mice, but this trend was not significant (Figure 5B).

Together, these experiments indicate that loss of RB does not significantly change the molecular or physical identity of MYC-induced HCC in this mouse model.

### Activation of *MYC* and deletion of Rb together lead to increased polyploidy in adult liver cells

We next investigated the potential effects of MYC activation and *Rb* deletion on the DNA content of hepatocytes in pre-neoplastic livers. Previous studies have reported that both overexpression of MYC alone and inactivation of RB alone in adult mice leads to an increase in ploidy of hepatocytes [44], [47], [48], [49], [50]. The histopathological analysis of liver sections from *Rb* and *MYC/Rb* mice after 5 months of induction of the *MYC* transgene and deletion of *Rb* showed accumulation of hepatocytes with large nuclei compared to control mice, before the appearance of tumors (Figure 5C).

FACS analysis (Figure 5D) showed that activation of MYC resulted in a non-significant increase in ploidy in hepatocytes after 5 months in this system, presumably because full MYC stabilization and activation is only effective once tumorigenesis has been initiated in cells [32], [39], [42] (DWF, unpublished observations). Loss of RB led to decreased numbers of 4n hepatocytes and a concomitant increase of 8n and 16n cells, consistent with increased polyploidy. Activation of MYC in *Rb* deficient mice also generated significantly more 4n hepatocytes compared to loss of RB alone. *MYC/Rb* double mutant mice showed a significant increase in ploidy compared to *MYC* mutant mice, with fewer 2n populations and more 8n and 16n populations (Figure 5D). These observations suggest that loss of RB may be more influential than activation of MYC in perturbing DNA content in adult hepatocytes, but also that the two genetic events cooperate to control endoreduplication of mature hepatocytes under these experimental conditions.

### *Rb/MYC* double mutant mice die from HCC faster than *MYC* mutant mice

*MYC* and *MYC/Rb* mutant mice develop tumors that are histopathologically similar and express comparable levels of cell cycle regulators. However, it was still possible that loss of RB function and activation of MYC may cooperate during tumor development; in particular, *MYC/Rb* mutant mice have more polyploid hepatocytes compared to *MYC* mutant mice before tumors are detectable (Figure 5D) and polyploidy has been associated with cancer, including liver cancer [51], [52]. We tested this possibility by monitoring the survival of *MYC* and *MYC/Rb* mutant mice over a year after activation of MYC and deletion of *Rb*. As reported before, not all *MYC* mutant mice develop HCC when the transgene is induced in adult mice [32], [42]. Indeed, we found that some mutant mice were still alive at 52 weeks and that none of these mice died from HCC between 52 and 75 weeks. Because mice tend to develop tumors naturally after one year of age, including some liver lesions, we focused our analysis on mice aged one year after the removal of doxycycline. Importantly, none of the control mice (wild-type for *MYC* and wild-type or mutant for *Rb*, n = 12) died from liver cancer within this first year (data not shown).

**Figure 6:**
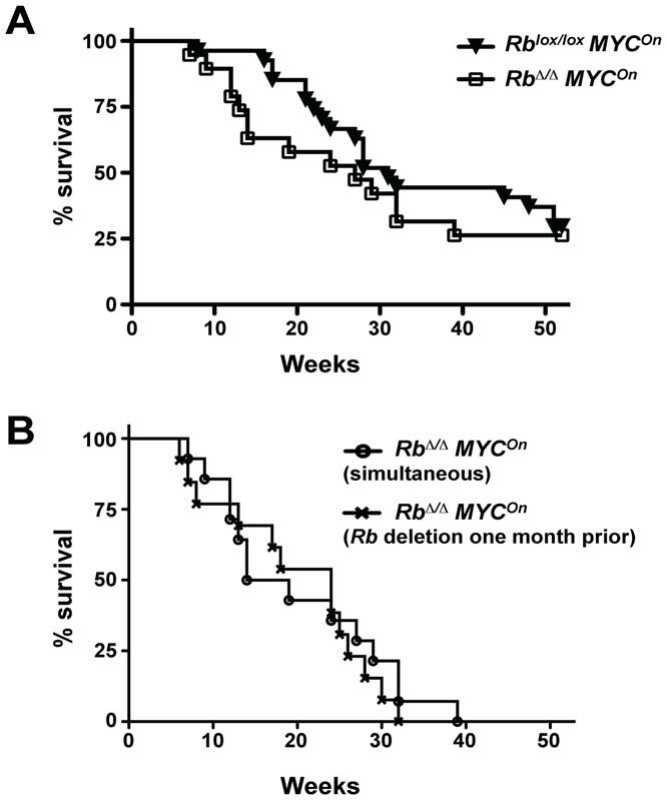
Survival analysis of Myc and Myc/Rb mutant mice. **A.** Kaplan-Meier survival analysis of *MYC* (n = 27) and *MYC/Rb* (n = 19) mutant mice when both tumorigenic events were triggered simultaneously (time 0) in adult mice (8–12 weeks after birth) by removal of doxycycline and Ad-Cre infection. **B.** Survival analysis of *MYC/Rb* mutant mice developing HCC when both tumorigenic events were triggered simultaneously in adult mice (8–12 weeks after birth) (n = 19) and when *Rb* deletion by Ad-Cre was performed 4 weeks before MYC activation (8–12 weeks and 12–16 weeks after birth, respectively) (n = 15); time 0 is the time of MYC activation.

We found that loss of *Rb* did not change the number of mice that do not develop HCC within one year, suggesting that *Rb* deletion does not affect cancer initiation and confirming that tumorigenesis is driven by activation of MYC in this mouse model (Figure 6A). *MYC* mutant mice had a median survival of 31 weeks while the median survival of *MYC/Rb* mutant mice was 27 weeks; when the mice that did not die from liver cancer were excluded from the analysis, the median survival for *MYC* and *MYC/Rb* mutant mice decreased to 27 and 16.5 weeks, respectively, with a P value of 0.07 in a Gehan-Breslow-Wilcoxon test. Thus, while not statistically significant, this analysis is indicative of a trend that *MYC/Rb* mutant mice die faster from HCC than mice with MYC activation alone, suggesting that loss of *Rb* may cooperate with activation of MYC in HCC.

The possibility to control MYC activation and *Rb* deletion with two different methods (removal of doxycycline and Cre-mediated recombination, respectively) also allowed us to test the possibility that the order of the mutations in this system may influence tumor development. While deletion of *Rb* is not sufficient to initiate cancer, it results in increased proliferation and ploidy. We hypothesized that activation of MYC in this context may facilitate and accelerate tumor development in the liver. To test this idea, we infected mice with Ad-Cre and then, one month later, activated MYC by removing doxycycline from the water. The analysis of these two cohorts of *MYC/Rb* mutant mice showed no difference in survival curves when survival was measured starting at the time of MYC activation (Figure 6B). Thus, in this context, pre-deletion of *Rb* does not affect tumor development in the liver of mice expressing MYC.

## Discussion

In this study we investigated if loss of RB function may cooperate with activation of MYC in a mouse model of HCC. While RB and MYC are important players in the tumorigenic process, this is the first time, to our knowledge, that their functional interactions have been tested genetically *in vivo*. We found that *MYC* and *MYC/Rb* mutant tumors closely resembled each other, including similar appearances grossly, histologically, and molecularly. The only identifiable difference was the degree of ploidy in pre-neoplastic tissue where *MYC/Rb* mutant pre-tumor tissue displayed a higher degree of ploidy than *MYC* mutant pre-tumor tissue in hepatocyte populations.

Several oncogenes and growth factors, such as E2F1 and TGFα, have been shown to cooperate with MYC in mouse models of HCC development [53], [54], [55], [56]. Clearly, tumors induced by oncogenes such as MYC must have some levels of inactivation of the RB pathway to be able to grow. However, it is surprising that we did not observe a strong enhancement of HCC development when *Rb* deletion is added to MYC activation, especially given the observation that many human samples of HCC tend to harbor mutations that simultaneously activate MYC and inactivate the RB pathway [57], [58]. There are a few possibilities that could explain this observation. For example, MYC has been shown to directly induce transcription of Cyclin D2 and Cdk4, which in turn leads to the sequestration of p27 away from Cyclin E and thus the accumulation of Cyclin E/Cdk2 complexes; these kinases then lead to the phosphorylation and inactivation of RB and its family members p107 and p130 [20], [21], [22]. In fact, inactivation of MYC delays phosphorylation of RB after growth stimulation [59]. Moreover, MYC induces the expression of miRNA genes that can repress the expression levels of *Rb* and other cell cycle inhibitors [60]. MYC can also directly activate genes downstream of RB/E2F complexes [61], [62], [63]. As a result, it is possible that MYC activation alone is enough to critically inactive the RB pathway. In support of this hypothesis, we found that cells expressing high levels of MYC also express lower levels of *Rb* mRNA compared to control hepatocytes (data not shown). However, as mentioned above, the observation that activation of MYC and loss RB function are selected for in many cancers, including HCC [20], [21], [22], [26], would argue against the idea that activation of MYC is sufficient to inactivate RB function and that loss of both *RB* alleles is merely a passenger effect.

Another possibility to explain the absence of strong cooperation between activation of MYC and deletion of *Rb* in our mouse HCC model is that mouse tumors driven by MYC overexpression develop by different mechanisms than human tumors expressing high levels of MYC, which would make loss of RB function dispensable. Thus, it will be interesting in the future to examine the consequences of deleting *Rb* in mice in which MYC activation is achieved using different systems, including different liver promoters and at different time points during tumorigenesis.

It is also possible that, similar to what has been observed in the eye of mice [64], the RB family members p107 and p130 have a stronger overlapping role in the mouse liver compared to the human liver. The idea that inactivation of several RB family members may be required to observe a strong phenotype during HCC development is supported by the observation that most of the events known to inactivate the RB pathway are upstream events and may inactivate RB, p107, and p130 simultaneously [65], [66], [67], [68], [69], [70], [71], [72], [73]. Future experiments may examine HCC development in *MYC/Rb* mutant mice in which *p107* and or *p130* are also inactivated, as well as the tumor phenotypes of mice in which *Rb* is deleted and MYC is activated in other tissues and organs. In fact, we have previously conducted a similar study examining the interaction between oncogenic RAS and loss of *Rb* in a mouse model of lung adenocarcinoma. In this model, RAS-induced tumors also showed partial inactivation of RB function, but loss of *Rb* had two effects: initially it increased cancer initiation and then led to decreased proliferation, presumably due to compensation by p107 and p130 [74]. As a result it appears that the effects of cooperation between an oncogene and a tumor suppressor may not be reliably predicted when based solely on the degree of overlap between their pathways.

Recent observations indicate that the timing of p53 re-expression during lung cancer development may affect cancer outcome [75], [76]. The system we employed utilizes the tetracycline-dependent regulatory system to activate MYC and the Cre-lox conditional mutation system to delete *Rb*. This enabled us to investigate the consequences of changing the time of the tumorigenic events. We hypothesized that prior mutation in the *Rb* gene may facilitate MYC-induced tumor development in the liver but found no differences in HCC development whether *Rb* was deleted before MYC was activated or at the same time. We also found that deletion of *Rb* one month after activation of MYC did not impact survival (data not shown). These negative results may be due to the fact that, as discussed above, loss of RB may not strongly modify the tumor phenotype of MYC-expressing mice in our model. Evidence from HCC patients suggests that *RB* loss is a late event in HCC development [66], and it is possible that a similar strategy in a different mouse model may identify specific functions for RB during HCC progression, including during metastasis [66].

One such function for RB may be to protect genome integrity. Recent evidence indicates that, in addition to its role at the G1/S transition of the cell cycle, RB may also play a critical role to prevent chromosomal instability [77], [78], [79], [80]. While the role of polyploidy in cancer development is still controversial, increasing evidence suggests that it may result in aneuploidy, which may contribute to cancer initiation [51], [52], [81], [82]. We found that loss of RB may increase polyploidy in MYC-expressing hepatocytes. Recent evidence also suggests that one mechanism of HCC development upon infection with hepatitis C virus (HCV) may be through increased genomic instability following inactivation of RB [48]. One limitation of our study is that the cell of origin of the liver tumors developing upon ectopic expression of MYC is unknown, thereby limiting any further investigation of the potential role of increased polyploidy in HCC development. It will be interesting in the future to determine in other cancer models if activation of MYC and loss of RB cooperate in HCC development by altering chromosome numbers.

## Materials and Methods

### Mice

All animal work was approved by Stanford IACUC committee (protocol number 13565) and follows AAALAC guidelines. TRE-*MYC* LAP-*tTA* mice were bred to either conditional *Rb^lox/lox^* or *Rb^lox/lox^ Rosa26-CreER* mice. The human *MYC* transgene was activated by the removal of doxycycline (100 µg/ml) from the drinking water. Recombination of alleles in *Rb^lox/lox^* mice was accomplished by splenic injection of 5×10^8^ pfu Ad-Cre (Vector Development Laboratory, Baylor). Control mice were treated with equal amounts of AdGFP (Baylor College of Medicine). The Cre recombinase was induced in *Rb^lox/lox^ Rosa26-CreER* mice by five consecutive daily injections of 1 mg of tamoxifen (Sigma) in corn oil; control mice were injected with corn oil alone [83]. All treatments were performed on adult mice (8–12 weeks old). Deletion of the *Rb* gene was monitored by genomic PCR, as described before [83].

### RNA analysis

RNA was extracted with TRIzol (Invitrogen) and cleaned with the RNeasy Mini Kit (QIAGEN). RT-PCR and quantitative Real-Time PCR were performed using the DyNAmo cDNA synthesis kit and the SybrGreenER Mastermix (Invitrogen), respectively. Primer Sequences: *Rb delta* forward 5′- GGAGAAAGTTTCATCCGTGGAT -3′ reverse 5′- GTGAATGGCATCTCATCTAGATCAA -3′; human *MYC* forward 5′-TGCTCCATGAGGAGACACC-3′ reverse 5′-CCTCATCTTCTTGTTCCTCCA-3′; *p107* forward 5′- CCGAAGCCCTGGATGACTT-3′ reverse 5′- GCATGCCAGCCAGTGTATAACTT-3′; *p130* forward 5′- TGTCCGGCCTCAGGAATG-3′ reverse 5′- CTGTCAGCGATAGCCTGAGTTG-3′; *p53* forward 5′- GCCCATGCTACAGAGGAGTC-3′ reverse 5′- AGACTGGCCCTTCTTGGTCT-3′; *p21* forward 5′-CAGATCCACAGCGATATCCA-3′ reverse 5′-GGCACACTTGCTCCTGTG-3′; *E2f1* forward 5′-TGCCAAGAAGTCCAAGAATCA-3′ reverse 5′-CTTCAAGCCGCTTACCAATC-3′; *Cyclin E1* forward 5′- CTGAGAGATGAGCACTTTCTGC-3′ reverse 5′- GAGCTTATAGACTTCGCACACCT-3′; *B-myb* forward 5′-TTACGCCGTACGTGGAAGA-3′ reverse 5′-TTCCAGTCTTGCTGTCCAAA-3′; *cdc25c* forward 5′- GGAAACACCCGGATCTGAA-3′ reverse 5′- ACTTTCCAGACAGCAAAGCAG-3′; *Cyclin A2* forward 5′-CTTGGCTGCCACCAACAGTAA-3′ reverse 5′-CAAACTCAGTTCTCCCAAAAACA-3′; *GAPDH* forward 5′-GGGTTCCTATAAATACGGACTGC-3′ reverse 5′-CCATTTTGTCTACGGGACGA-3′; *Afp* forward 5′- TGCTGCAAAGCTGAAAATGC -3′ reverse 5′- GCTGCTTTCTCTTAATTCTTTTGTAACTG -3′; *Albumin* forward 5′-AGTGTTGTGCAGAGGCTGAC-3′ reverse 5′-TTCTCCTTCACACCATCAAGC-3′; *CK19* forward 5′-TGACCTTGGAGATGCAGATTG-3′ reverse 5′-CCTCAGGGCAGTAATTTCCTC-3′.

### Immunoblot analysis

Whole cell extracts from tissue were prepared in 50 µM Tris pH 7.4, 250 µM NaCl, 10% glycerol, 0.5% Triton-X-100, and a cocktail of protease inhibitors. 75 µg of protein extract were run on acrylamide gels and transferred to a PVDF membrane (Hybond). Membranes were probed with the following antibodies: CDK2 (Santa Cruz Biotechnology, sc-163-G), Cyclin E1 (Santa Cruz Biotechnology, sc-198), E2F1 (Santa Cruz Biotechnology, sc-193X), p107 (Santa Cruz Biotechnology, sc-318), p53 (Vector Laboratories, CM5), PCNA (Santa Cruz Biotechnology, PC10), and β-Actin (Sigma, A5441). Secondary antibodies conjugated to HRP (Jackson ImmunoResearch) were used at a concentration of 1∶5000, and signal was detected by ECL (Invitrogen).

### Histology and Immunostaining

Mouse organs were fixed in 4% paraformaldehyde overnight and embedded in paraffin. Sections were stained with hematoxylin and eosin (H&E). The histopathological analysis was performed blind by a trained pathologist (H.V.). Mice were injected with 1 mg of BrdU (Sigma) 4 hours prior to dissection. Sections were dewaxed and rehydrated in Trilogy buffer (Cell Marque) in a pressure cooker for 15 minutes, blocked in 5% serum for 30 min, and incubated with primary antibody overnight at 4°C. The next day, sections were incubated with secondary antibody for 1 hour, stained with DAPI (for immunofluorescence) or hematoxylin (for immunohistochemistry), and mounted. Antibodies used include Ki67 (Becton-Dickinson, 550609), BrdU (Becton-Dickinson, 347580), and Cleaved Caspase-3 (CC3, Cell Signaling, 96645). Quantification of cell cycle and cell death was performed using the BioQuant imaging analysis software.

### Flow cytometry

Hepatocyte nuclei were extracted by grinding murine liver in Lysis Buffer (25 mM Tris pH 7.5, 50 mM KCl, 2 mM MgCl_2_, 1 mM EDTA, and 1 mM PMSF), followed by homogenization and centrifugation at 12,000 rpm for 30 seconds [49]. Pellets were then resuspended in Lysis Buffer and centrifuged again at 12,000 rpm for 30 seconds. These pellets were then resuspended in PI Buffer (0.5 mg/ml Propidium Iodide, 0.1% NP-40, 0.1% Sodium Citrate, 40 µg/ml RNase A, in PBS) for 30 min. FACS analysis was performed with a BD FACSCalibur instrument and data was analyzed using the FlowJo software (Tree Star).

### Statistical analyses

Statistical significance was assayed by Student's t-test using the GraphPad Prism software, except for Figure 5D where ANOVA was used to compare the four genotypes for each ploidy analyzed and for Figure 6 where the Gehan-Breslow-Wilcoxon test was used to compare the survival of mouse cohorts. Mean and standard error are shown. ns, not statistically significant, *, p<0.05 - ** p<0.005 - ***, p<0.001.
